# Regarding: “the impact of body mass index on the efficacy of CDK4/6 inhibitors in patients with metastatic breast cancer”

**DOI:** 10.1080/07853890.2026.2620175

**Published:** 2026-02-01

**Authors:** Dong Li, Cong Sun, Chang Wang

**Affiliations:** a Department of Breast and Thyroid Surgery, Zibo First Hospital, Zibo, Shandong, P.R. China; b Department of Gynecology, Zibo First Hospital, Zibo, Shandong, P.R. China; c Department of General Surgery, Wenshang County People’s Hospital, Jining, Shandong, P.R. China

**Keywords:** Body mass index, CDK4/6 inhibitors, metastatic breast cancer, hormone receptor–positive, treatment efficacy

Dear Editor

We read with great interest the article by Ma et al. investigating the relationship between BMI and survival outcomes in HR-positive metastatic breast cancer patients treated with CDK4/6 inhibitors [1]. The authors report that lower BMI is associates with poorer overall survival in first-line treatment settings, challenging traditional assumptions about obesity and cancer prognosis. While this work contributes valuable real-world data to an area of ongoing debate, we wish to highlight several methodological considerations that may influence the interpretation of these findings.

A primary concern relates to treatment intensity, which remains unexamined in the current analysis. The PALLAS trial demonstrated that high-BMI patients experience fewer CDK4/6 inhibitor-related adverse events [2], suggesting the survival advantage may reflect superior treatment tolerance rather than metabolic protection. Recent evidence confirms that early dose reductions significantly compromise survival outcomes [3]. Without data on relative dose intensity and treatment discontinuation stratified by BMI group, it remains unclear whether BMI functions as an independent prognostic factor or merely as a surrogate for treatment tolerability.

We also note concerns regarding survival estimation methodology. The authors report a mean overall survival of 78.9 months for the high-BMI group when median survival was not reached. Under conditions of substantial censoring, mean survival estimates may be unstable and sensitive to distributional assumptions in the data tail. Recent methodological advances emphasize restricted mean survival time as a more robust alternative when median survival cannot be reliably estimated [4]. Combined with the modest 20-month median follow-up and borderline statistical significance (*p* = 0.047), this approach may not fully capture the reliability of the observed difference.

Related to this, the exclusion of tumor markers from multivariate models merits reconsideration. While the authors justify removing CEA, CA125, and CA153 based on hazard ratios approximating unity, this decision overlooks potential clinical relevance. Notably, CEA levels differ significantly between BMI groups (*p* = 0.010), with higher values in low-BMI patients. Multiple studies demonstrate that elevated CEA reflects greater tumor burden and independently predicts poorer survival in metastatic breast cancer [5]. Rather than excluding these variables, mediation analysis could clarify whether BMI effects operate through disease burden pathways, thereby strengthening causal inference.

The temporal relationship between BMI measurement and disease course warrants further examination. BMI assessed at CDK4/6 initiation conflates constitutional body habitus with disease-induced weight changes. For late-line patients, low BMI may reflect disease consequence rather than baseline predictor. Additionally, the “first-line” cohort includes both de novo stage IV and recurrent disease patients with distinct prognoses. Stratification by disease presentation would reduce this heterogeneity and strengthen the analysis.

These observations represent opportunities to deepen mechanistic insight into BMI’s complex role in CDK4/6 inhibitor therapy. Future investigations incorporating treatment intensity metrics, longitudinal anthropometric assessments, and mediation analyses would clarify whether the obesity paradox reflects true biological protection or confounding by treatment tolerance and disease burden.

## Data Availability

There is no data associated with this research.
